# Targeting the gut microbiota to enhance the antitumor efficacy and attenuate the toxicity of CAR-T cell therapy: a new hope?

**DOI:** 10.3389/fimmu.2024.1362133

**Published:** 2024-03-15

**Authors:** Peng-Fei Zhang, Dan Xie

**Affiliations:** ^1^ Gastric Cancer Center, Division of Medical Oncology, Cancer Center, West China Hospital, Sichuan University, Chengdu, China; ^2^ Department of Medical Genetics, West China Second University Hospital, Sichuan University, Chengdu, China; ^3^ Key Laboratory of Birth Defects and Related Diseases of Women and Children (Sichuan University), Ministry of Education, Chengdu, China

**Keywords:** gut microbiota, CAR-T cell therapy, immunotherapy, antitumor efficacy, toxicity

## Abstract

Chimeric antigen receptor (CAR) -T cell therapy has achieved tremendous efficacy in the treatment of hematologic malignancies and represents a promising treatment regimen for cancer. Despite the striking response in patients with hematologic malignancies, most patients with solid tumors treated with CAR-T cells have a low response rate and experience major adverse effects, which indicates the need for biomarkers that can predict and improve clinical outcomes with future CAR-T cell treatments. Recently, the role of the gut microbiota in cancer therapy has been established, and growing evidence has suggested that gut microbiota signatures may be harnessed to personally predict therapeutic response or adverse effects in optimizing CAR-T cell therapy. In this review, we discuss current understanding of CAR-T cell therapy and the gut microbiota, and the interplay between the gut microbiota and CAR-T cell therapy. Above all, we highlight potential strategies and challenges in harnessing the gut microbiota as a predictor and modifier of CAR-T cell therapy efficacy while attenuating toxicity.

## Introduction

Immunotherapy, which includes a variety of approaches, such as tumor vaccines, immune checkpoint inhibitors (ICIs), and adoptive cell therapy (ACT), has made tremendous advances in cancer treatment in recent decades (1–3). Chimeric antigen receptor T (CAR-T) cell therapy, as the most concerned treatment in immunotherapy, has impacted the history of cancer therapy and attracted increasing amount of attention worldwide (4). CARs are versatile synthetic receptors that are genetically engineered to express in T cells. CARs comprise an ectodomain with an antigen-binding module and a hinge, a transmembrane region, and costimulatory/activation domains (such as the signaling domains of CD3ζ, CD28 and/or CD137) (5, 6). Currently, CAR-T cell therapy is being increasingly studied in a variety of tumors, especially in patients with recurrent/refractory (R/R) B cell leukemia or lymphoma (7–10). To date, a series of CAR-T cell products, such as Kymriah, Yescarta, Tecartus and Breyanzi, have been approved for the treatment of R/R B cell leukemia or lymphoma (11–14). The unprecedented success of CAR-T cell therapy in treating hematological malignancies has sparked interest in broadening the application of CAR-T cell therapy in solid tumors. Currently, more than 200 clinical trials investigating CAR-T cells for the treatment of solid tumors have been launched around the world; however, the results of applying CAR-T cells to solid tumors have been disappointing. In most studies, only a few patients achieved partial response (PR) (15, 16). In addition to poor efficacy in solid tumors, several other disadvantages also limit the wide application of CAR-T cells. CAR-T cells therapy is often accompanied by self-reactivity mediated by on-target, off-tumor antigen expression, increased systemic inflammation leading to cytokine release syndrome (CRS), neurotoxicity, and suppression of humoral immunity due to B-cell aplasia (17–19). These disadvantages limit CAR-T cells from reaching their full therapeutic potential and therefore highlight the critical need for biomarkers that can predict and improve clinical outcomes with future CAR-T cell treatments.

The gut microbiota refers to the vast collection of microbes living in the gastrointestinal tract (20). With the development and wide application of novel molecular technologies (such as 16S ribosomal RNA sequencing, metagenomics and metabolomics), a multitude of preclinical and clinical studies have been performed to explore intricate host-microbiota interactions over the last decade (21). First, the gut microbiota plays a key role in several physiological processes, including immunity, metabolism, and the inflammatory response (22, 23). Second, gastrointestinal tract dysbiosis promotes the occurrence and progression of a variety of malignant tumors (24). Third, the gut microbiota has been demonstrated to affect the response to several anticancer therapeutics, such as chemotherapy, radiotherapy, and immune checkpoint blockade (ICB) (25, 26). In light of these findings, there is emerging interest in investigating the role of the gut microbiota in CAR-T cell therapy. For example, in a recent study, Smith et al. investigated the influence of the gut microbiota on the response and toxicity of hematologic malignancies to CD19 CAR-T cell therapy. A total of 228 patients treated with CD19 CAR-T cells were recruited and stratified based on exposure to broad-spectrum antibiotics, including piperacillin, imipenem or meropenem (PIM), within four weeks prior to the first treatment with CAR-T cells. Strikingly, patients exposed to broad-spectrum antibiotics had significantly shorter overall survival (OS), an increased incidence of immune effector cell-associated neurotoxicity syndrome (ICANS) and a trend toward an increased incidence of CRS compared to patients without broad-spectrum antibiotic exposure (27). In this review, we summarize the general aspects of CAR-T cell therapy and the gut microbiota, focus on the current understanding of the interplay between the gut microbiota and CAR-T cell therapy, and highlight the potential of microbiota-based prognostic prediction and gut microbiota-based biotherapy to potentiate CAR-T cell efficacy while attenuating toxicity.

## CAR-T cell therapy

In recent years, CAR-T cell therapy has shown unprecedented antitumor efficacy and stands at the novel forefront of current cancer therapy (28). CAR-T cell therapy involves a series of complex processes, including genetically modified T cells to express CAR toward a tumor-specific antigen (TSA) or tumor-associated antigen (TAA) via viral and non-viral transfection methods. After *in vitro* T-cell amplification, CAR-T cells will be reinfused back into patients to eradicate tumors (29). CARs are synthetic receptors, and the basic structure of CARs contains an extracellular antigen-binding region that binds to the antigen (usually a single-chain variable fragment (scFv) of an antibody), a hinge region providing flexibility, a transmembrane region and an intracellular signal transduction region that activates the cytotoxic functions of CAR-T cells upon antigen recognition. Over the past decade, the design of CARs has progressed rapidly. To date, fourth generation CARs have been developed based on different intracellular signals (30). First-generation CARs contain only one CD3ζ signaling domain to stimulate cytotoxic CAR-T cell activity, which mimics the process of natural biologic T-cell activation. However, the clinical efficacy of first-generation CAR-T cells has been disappointing in the majority of early trials because of the short persistence and low expansion ability of CAR-T cells *in vivo* (31, 32). To solve this problem, second-generation CARs, which contain an additional costimulatory molecule, such as CD28, OX40 or 4-1BB, have been developed. The addition of a costimulatory signal domain can recapitulate the natural costimulation of T cells and significantly improve the antitumor ability of CAR-T cells (33, 34). To further enhance the antitumor effect of CAR-T cells, third-generation CARs that combine multiple costimulatory molecules have also been developed (35, 36). Fourth-generation CARs are designed to express more powerful weapons, such as cytokines, receptors for chemokines, and a controlled suicide gene. Compared with second- and third-generation CAR-T cells, fourth-generation CAR-T cells exhibit rapid expansion, high tumor killing activity, and obvious advantages in terms of safety and persistence (37–39). In the past decade, CAR-T cell therapy has shown remarkable efficacy in the treatment of patients with R/R hematological malignancies (7–10). Thus, several CAR-T cell medicines, such as CD19-targeted Tisagenlecleucel (Kymriah®), Axicabtagene ciloleucel (Yescarta®), brexucabtagene autoleucel (Tecartus®), lisocabtagene maraleucel (Breyanzi®), B cell maturation antigen (BCMA)-targeted idecabtagene vicleucel (Abecma®), and ciltacabtagene autoleucel (Carvykti®), have been approved by the U.S. Food and Drug Administration (FDA) and European Medicines Agency (EMA) for the treatment of hematological malignancies, including lymphomas, some forms of leukemia, and most recently for the treatment of multiple myeloma (MM) (11–14, 40, 41).

Inspired by the tremendous success of CAR-T cell therapy in hematological malignancies, researchers are gradually expanding the application of CAR-T cell therapy to the treatment of solid tumors. Over the years, a series of CAR-T cells targeting antigens in solid tumor cells, including carcinoembryonic antigen (CEA), epidermal growth factor receptor (EGFR), human epidermal growth factor receptor 2 (HER2), and prostate-specific membrane antigen (PSMA), have been investigated in preclinical and clinical settings (42–48). However, the antitumor efficacy of CAR-T cell therapy in these solid tumor settings has been disappointing. Several factors have impeded the utility of CAR-T cell therapy for solid tumors, including barriers that inhibit the trafficking and infiltration of CAR-T cells into the tumor, lack of specific tumor antigens that are highly and uniformly expressed in tumor cells, and hostile tumor microenvironment (TME) characterized by oxidative stress, nutritional depletion, acidic pH, and hypoxia (15, 16, 49, 50). Therefore, biomarkers for identifying the favorable prognostic patients receiving CAR-T cells are urgently needed.

## CAR-T cell therapy-related toxicity

In addition to the low response or high relapse rate in patients treated with CAR-T cells, treatment with CAR-T cells also shows severe adverse events (AEs), which include CRS, ICANS, cardiotoxicity, hypersensitivity reactions, fatal macrophage activation syndrome (MAS), and uveitis (17). These fatal AEs result in doctors walking on thin ice when prescribing CAR-T cells. CRS, which is defined as an excessive release of cytokines, such as interleukin (IL)-1, IL-6, interferon (IFN)-γ, and IL-10, following the activation of immune cells during immunotherapy, represents the most common AE associated with CAR-T cell therapy and occurs in 40–93% of patients receiving CAR-T cell therapy (51). CRS usually occurs within 1-2 weeks after CAR-T cells administration (52, 53). Common symptoms of CRS include fever, exhaustion, anorexia, myalgia, and arthralgia. CRS remains a major hurdle for the widespread use of CAR-T cells, if not properly identified and managed, CRS can be fatal and further progresses to more severe forms of the syndrome, including cardiac arrhythmia, tachycardia, and respiratory or multi-organ conditions (54, 55). Another severe AE in patients treated with CAR-T cells is ICANS, which occurs in up to 67% of leukemia patients and 62% of lymphoma patients (17). Impaired cognition and overall confusion, such as aphagia, lethargy, and delirium, represent the initial symptoms of ICANS (56, 57). With the progression of the disease, symptoms, including hallucinations, encephalopathy, seizures, and cerebral edema, can be observed in patients treated with CAR-T cells (58). Although the exact causes of ICANS are not fully understood, blood brain barrier disruptions, the influx of cytokines into the central nervous system (CNS), and microglial and myeloid activation within the CNS are regarded as key factors in the development of ICANS (59–61). Although a series of treatment options, including intravenous hydration corticosteroids, monoclonal antibodies against the IL-6 receptor, IL-1 receptor antagonist, or targeting granulocyte-macrophage colony-stimulating factor (GM-CSF), have been approved to relieve these AEs, toxicity is still a major challenge for the wide application of CAR-T cells. Therefore, methods for assessing and predicting potential toxicities before administration are also urgently needed (62).

## The gut microbiome and cancer

The gut microbiota, which refers to the vast collection of microbes inhabiting in the gastrointestinal tract, is involved in several key processes of human health, including providing nutrients and vitamins, protecting against pathogens, helping the development of the immune system and maintaining epithelial mucosa homeostasis (22, 23, 63). The balance between human cells and the gut microbiota is critical for maintaining the host’s health status. The gut microbiota can be modulated through several methods, including consuming dietary fibers, taking probiotics, or performing physical activity (64–68). In contrast, antibiotics, proton pump inhibitors (PPIs), smoking, and chronic stress may impair bacterial variability (69–72). Recently, several studies have suggested that the gut microbiota of cancer patients is very different from that of healthy individuals, and the role of the gut microbiota as a contributor to carcinogenesis has been well studied (73, 74). To date, the exact mechanisms through which the gut microbiota contributes to oncogenesis are not fully understood; however, several possible mechanisms can account for the process: (1) direct oncogenic effects of microorganisms and their products; (2) increased circulating pro-carcinogenic metabolites; and (3) disruption of cancer immunosurveillance through pro-inflammatory and immunosuppressive pathways (75).

**Figure 1:**
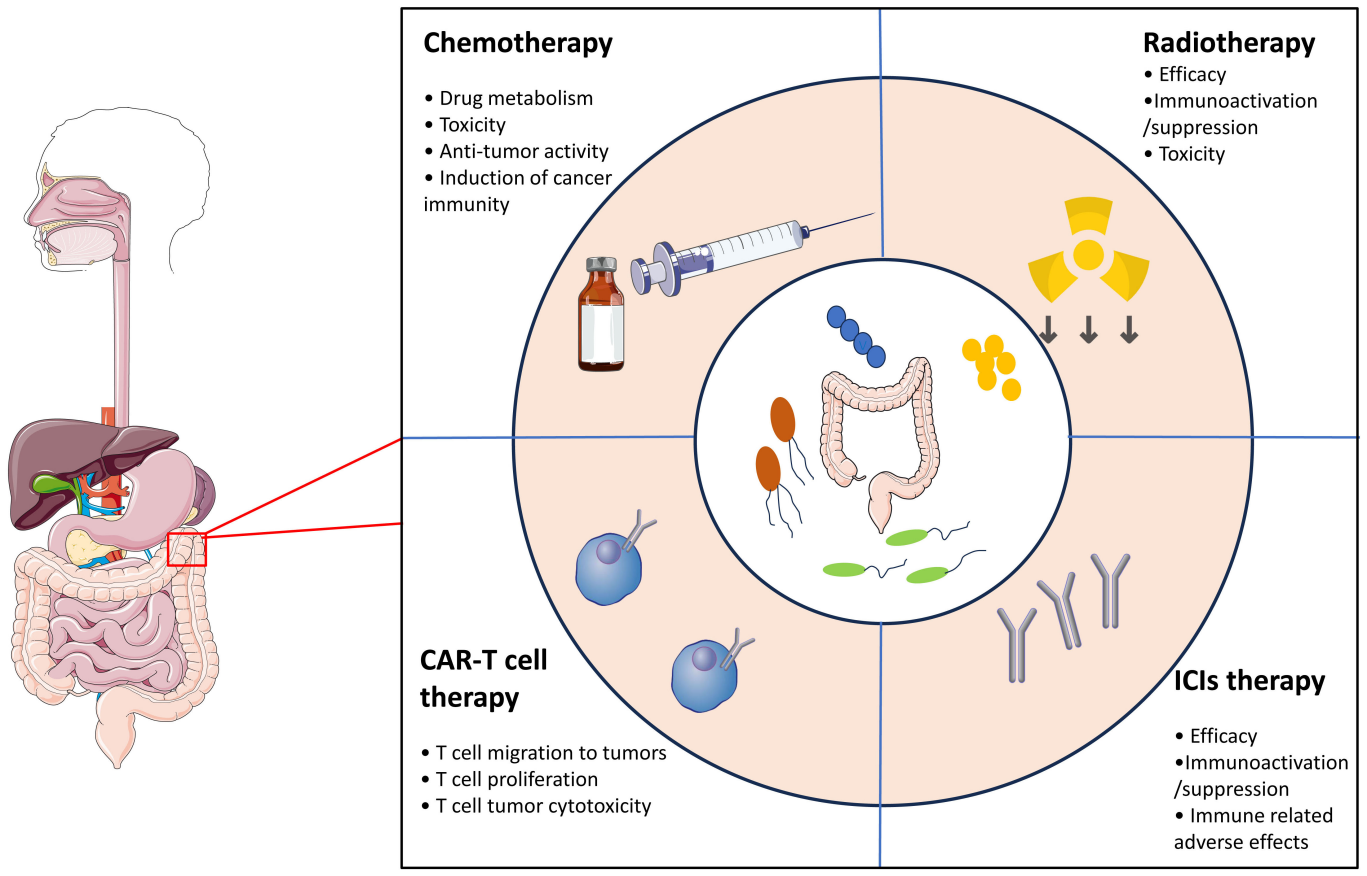
The gut microbiota modulates the response and susceptibility to side effects in response to different therapeutic strategies.

In addition to its pro-carcinogenic properties, recent preclinical and few clinical studies suggest that the gut microbiota can modulate the response and susceptibility to side effects of different therapeutic strategies, including surgery, chemotherapy, radiotherapy and immunotherapy (76, 77) (**Figure 1**). Although the underlying mechanisms are not well understood, some of them have been described as TIMER framework mechanisms, including translocation, immunomodulation, metabolism, enzymatic degradation and reduced diversity (78, 79). In this section, we summarize the role of the gut microbiota in modulating the efficacy, resistance and toxicity of cancer therapies.

### Chemotherapy

Recently, the gut microbiota has been demonstrated to be a key factor affecting the efficacy, pharmacokinetics, and toxicity of chemotherapy (80, 81). To date, a series of mechanisms, including xenometabolism, immune interactions, and altered community structure, have been proposed to account for the impact of the gut microbiota on chemotherapy efficacy (79). The gut microbiota can directly modify the metabolism of antitumor drugs, which is linked to an increase in toxicity and a decrease in treatment efficacy (82, 83). For example, the toxicity of irinotecan, a topoisomerase I inhibitor widely used to treat colorectal cancer, is dependent on glucuronosyltransferases in the liver. The active form of irinotecan, SN-38, can be detoxified into the inactive form SN-38-G by glucuronosyltransferases (84). However, in a *in vivo* rat model, *Bacteroides species* and other bacteria that express β-glucuronidase (such as *Faecalibacterium prausnitzii* and *Clostridum* spp.), can convert SN-38-G back into SN-38 (85). This process is associated with the accumulation of SN-38 in the gut, which leads to diarrhea. In addition, toxicity is preventable with broad-spectrum antibiotics or inhibitors of β-glucuronidase in *in vivo* studies (86). On the other hand, dysbiosis of the gut microbiota have also been suggested to be correlated with severe side effects, such as intestinal mucositis induced by doxorubicin, 5-FU or irinotecan (87, 88). For example, several studies in mice have shown that 5-FU therapy induces intestinal dysbiosis by increasing the abundance of *Staphylococcus* and *Clostridium* species and decreasing the abundance of *Enterobacteriaceae*, *Lactobacillus* and *Bacteroides*, thus exacerbating severe side effects in murine cancer models and in cancer patients (89). In addition to its role in reducing the toxicity of chemotherapy, the efficacy of chemotherapy can also be modulated by the gut microbiota (90, 91). Possible mechanisms include remote immune modulations by the gut microbiota and/or bacterial translocation to lymphoid organs. Several studies have also suggested that the gut microbiota provides a tumor microenvironment that can favor the toxic effect of drugs on cancer cells and sustain anticancer adaptive immunity following drug-induced immunogenic cell death (84). In a pioneer *in vivo* study, Iida et al. reported that oxaliplatin and cisplatin had reduced antitumor efficacy and chemoresistance in germ-free or antibiotic-treated mice, compared to specific-pathogen-free (SPF) mice (92). The gut microbiota primes myeloid cells in tumors to produce reactive oxygen species (ROS) via NADPH oxidase 2 (NOX2), which are important for DNA damage and apoptosis in response to platinum compounds. In addition, several *in vivo* studies have demonstrated that cyclophosphamide (CTX) can modulate the immune microenvironment of tumors by reducing regulatory T cells (Tregs) and increasing Th1 and Th17 cells in a manner dependent on the gut microbiota (90, 91, 93). The translocation of *Enterococcus hirae* to lymph nodes and the accumulation of *Barnesiella intestinihominis* in the colon promoted cancer immunity following CTX treatment. Although further clinical studies are needed to evaluate these innovative findings, the gut microbiota has been demonstrated to be an essential biomarker for cancer chemotherapy efficacy.

### Radiotherapy

Radiotherapy, which directly induces DNA damage through the production of ROS or reactive nitrogen species (RNS), is another important treatment option for cancer patients (80, 94). Moreover, radiotherapy can also induce local immunogenic effects and stimulate the innate immune system (95, 96). However, the tumor response after radiotherapy is heterogeneous, and the causes of this heterogeneity remain unclear. Recently, growing evidence has suggested that the gut microbiota may contribute to the interpatient heterogeneity of radiotherapy. In a *in vivo* mouse study, Cui et al. investigated the role of the circadian rhythm in the effect of radiotherapy and reported that mice with a normal 12-h dark/12-h light cycle had significantly better survival than did those with different cycles (8-h dark/16-h light or 16-h light/8-h dark) (97). In this process, alteration of the gut microbiota was suggested to account for the radio-resistance. Moreover, several other studies have suggested that depletion of the gut microbiota by broad-spectrum antibiotics results in an expansion of the *Saccharomycetes* class of fungi, which decreases radiotherapy efficacy by inhibiting tumor immunity by signaling through the β-glucan receptor Dectin 1 (98). Another hypothesis concerns the link between radio-resistance and gut microbiota-involved autophagy regulation. In a cohort study, Digomann et al. reported that several autophagy-related proteins were correlated with the clinical prognosis of patients with head and neck squamous cell carcinoma treated with radiotherapy (99).

On the other hand, radiotherapy side effects alter quality of life and are integral parts of treatment decisions. The gut microbiota plays a key role in radio-induced toxicity. In a study, Ferreira et al. reported a close correlation between the gut microbiota abundances and radiation enteropathy (100). In patients with radiation enteropathy, the composition of *Clostridium*, *Roseburia* and *Phascolarctobacterium* were significantly increased. In another study, a significant alteration in the *Firmicutes*/*Bacteroidetes* ratio was observed in patients with pelvic radiation-induced diarrhea (101). In a mouse model, fecal transplantation improved gastrointestinal tract function in irradiated mice and protected against radiation-induced death (102). Taken together, these data suggested that the gut microbiota plays a key role in the modulation of radiosensitivity and radiation-induced toxicity. However, further preclinical and clinical studies are needed to determine the detailed underlying mechanisms involved.

### Immunotherapy

Immunotherapy, which includes ICB therapy that inhibits programmed cell death protein 1/programmed cell death ligand 1 (PD-1/PD-L1) and cytotoxic T lymphocyte-associated antigen-4 (CTLA-4) signaling, tumor vaccines, and ACT, represents a hotspot of cancer therapy (103). Despite the remarkable effectiveness of immunotherapy in a subset of patients, most patients treated with immunotherapy will experience primary or acquired resistance (104, 105). Furthermore, immune-related adverse events (irAEs) are another challenge that limits the wide application of immunotherapy (106). Recently, accumulating evidence has pinpointed the indispensable roles of the gut microbiota in cancer immunotherapy. In a murine melanoma model generated by Paulos et al., the antitumor efficacy of CD8+ T cells was strongly increased after total body irradiation through the translocation of gut bacteria into mesenteric lymph nodes (107). This effect could be attributed to the fact that irradiation induces the release of microbial lipopolysaccharide (LPS), which activates the innate immune response and enhances the efficacy of antitumor CD8+ T cells. Antibiotic treatment or LPS neutralization can significantly decrease the antitumor response. In another *in vivo* study, Iida et al. reported that the efficacy of anti-IL-10/CpG oligodeoxynucleotide (ODN) immunotherapy was impaired by antibiotic treatment in both murine models of MC38 colon carcinoma and B16 melanoma (92). Antibiotic treatment can decrease the gut microbiota load and the number of proinflammatory cytokine-producing monocytes in tumors, leading to failure of the immunotherapy response. All these studies highlighted the important role of the gut microbiota in the antitumor efficacy of immunotherapy.

After the relationship between the gut microbiota and immunotherapy was recognized, an increasing number of studies have been conducted to explore the impact of the gut microbiota on the efficacy and toxicity of ICI therapy. Although the underlying mechanisms are not well understood, growing evidence confirms the central role of remote lymphoid and myeloid cell modulation by the gut microbiota. In a study, GF mice or antibiotic-treated mice were not capable of responding to the CTLA-4 antibody compared to SPF mice (108). The gut microbiota, which includes *Bacteroides thetaiotaomicron*, *Bacteroides fragilis* and *Burkholderia cepacia*, was suggested to be associated with the antitumor efficacy and toxicity of anti-CTLA-4 inhibitors. After oral administration of *Bacteroides* spp., the antitumor efficacy of the anti-CTLA-4 inhibitor was restored, with an increase in intratumoral mature DCs and an increase in the Th1 response in tumor-draining lymph nodes. Moreover, the microbiota composition could predict the status of solid tumor responders and nonresponders to anti-PD-1/PD-L1 therapy. In a study conducted by Gopalakrishnan et al., enrichment of *Faecalibacterium species* in the gut microbiota of melanoma patients was associated with a high response to anti-PD-L1 therapy, while enrichment of *Bacteroides thetaiotaomicron*, *Escherichia coli*, and *Anaerotruncus coli hominis* was found mainly in nonresponders (109). Fecal microbiota transplantation (FMT) via responder patients into GF mice improved ICI efficacy, which was associated with increased numbers of intratumoral mature DCs, IFN-γ+CD8+ and/or CD4+ antitumor T cells and decreased numbers of intratumoral CD4+FoxP3+ Tregs (109–111). In total, these promising results strongly support the use of microbial targeting in antitumor immunotherapy to enhance tumor efficacy.

## Influence of the gut microbiota on the effectiveness of CAR-T cell therapy

Currently, CAR-T-cell therapy is one of the most promising cancer therapies and has led to unprecedented responses in patients with R/R hematologic malignancies, including lymphoma, leukemia, and MM. In the past decade, the correlation between the gut microbiota and the antitumor efficacy of CAR-T cells has been explored in a series of preclinical and clinical studies, and it is not unexpected that the gut microbiota represents a key factor in predicting and determining the outcomes of CAR-T-cell therapy. Smith et al. conducted the first human study to investigate the influence of the gut microbiota on the response and toxicity of CD19 CAR-T-cell-based therapies (27). A retrospective cohort of 228 patients from the Memorial Sloane Kettering Cancer Center (MSKCC) and University of Pennsylvania who were treated with second-generation CD19 CAR-T cells was established; 137 of these patients were non-Hodgkin lymphoma (NHL) patients, 91 of whom were acute lymphocytic leukemia (ALL) patients. Then, they analyzed the fecal microbiota composition of patients receiving CD19 CAR-T-cell therapy and hypothesized that the microbiota is associated with antitumor efficacy and toxicity. The baseline stool samples obtained prior to CAR-T-cell therapy were heterogeneous for bacteria at the phylum level, as indicated by a decreased Shannon index for alpha diversity compared to that of healthy controls. The authors stratified patients based on exposure to antibiotics, given that antibiotics are commonly used to treat secondary infections in these patients. Indeed, 60% of the patients received antibiotics, and 20.6% of them specifically received broad-spectrum antibiotics such as piperacillin/tazobactam, imipenem/cilastatin, and meropenem (PIM), which target anaerobic gut commensal bacteria. Using OS and progression-free survival (PFS) as indices of treatment response, the authors found that PIM exposure prior to CAR-T-cell therapy was correlated with worse OS and PFS. To further validate this hypothesis, the authors established a prospective cohort of 48 NHL or ALL patients with matched baseline gut microbiota profiles and found that response (measured as complete response (CR) on day 100 post treatment) to CAR-T-cell therapy was positively correlated with baseline microbiota diversity and enrichment of specific bacterial taxa, such as *Ruminococcus*, *Faecalibacterium*, and *Bacteroides*. In another recent study by Stein-Thoeringer et al., a large cohort of lymphoma patients receiving second-generation CD19 CAR-T cells (122 were treated with axicabtagene ciloleucel (axi-cel), 49 received tisagenlecleucel (tisa-cel) and 1 received lisocabtagene maraleucel (liso-cel)) in Germany and the U.S. were established (112). Consistent with the findings of previous studies, an association between exposure to antibiotics prior to CAR-T-cell infusion and increased incidences of cancer relapse/disease progression and a decrease in OS was also observed. Moreover, based on machine learning methods used to explore microbiome-based predictions of treatment outcomes, *Bacteroides, Ruminococcus, Eubacteria*, and *Akkermansia* spp. were identified as major potential drivers of therapy responsiveness. Similarly, Hu and colleagues also revealed significant differences in the enrichment of *Bifidobacterium*, *Prevotella*, *Sutterella*, and *Collinsella* between MM patients treated with second-generation BCMA CAR-T cells in complete remission and those in partial remission (113). However, in a study investigating how vancomycin-induced gut microbiota dysbiosis affects CAR-T-cell therapy, the results were different (114). Vancomycin is a branched tricyclic peptide antibiotic that targets mostly gram-positive bacteria. When administrated orally, vancomycin is poorly absorbed and does not reach an effective dose systemically due to its large size. In this study directed by Uribe-Herranz et al., mice receiving vancomycin in combination with murine CD19bbz CAR-T-cell therapy showed an increased tumor response and tumor-associated antigen (TAA) cross-presentation compared with those of mice receiving CD19 CAR-T-cell therapy alone, both in lymphoma and melanoma murine models. FMT from healthy human donors to preconditioned mice recapitulated the results obtained in naive gut microbiota mice. 16s Metagenomic analysis demonstrated that oral vancomycin affected the composition of human gut microbiome of engrafted avatar mice, with a significant decrease of alpha diversity and significant expansion of vancomycin-resistant bacteria (such as *Enterobacteriaceae* and *Sutterellaceae*). The decrease or depletion of gram-positive bacteria included several families, genera, and species, including *Ruminococcaceae* and *Lachnospiraceae*, which are known to impair antigen presentation. Similarly, under clinical conditions, compared with unexposed patients, B-ALL patients treated with CD19 CAR-T cells and exposed to oral vancomycin had greater CAR-T-cell peak expansion. Taken together, although research on the role of the gut microbiota in CAR-T-cell therapy is still in its infancy, these findings suggest the tremendous potential of the gut microbiota as a noninvasive prognostic marker for CAR-T-cell therapy.

## Interactions between the gut microbiota and toxicities related to CAR-T cell therapy

In addition to the effect of the gut microbiota on the antitumor efficacy of CAR-T cells, the gut microbiota was also demonstrated to alter the development of toxicity in patients treated with CAR-T cells. Smith and colleagues investigated the role of the gut microbiota in the toxicity of CAR-T cells in a multicenter study of patients with B-cell lymphoma and leukemia (27). In a retrospective cohort (n= 228), they found that exposure to antibiotics, particularly PIM, was associated with shorter survival and increased neurotoxicity. Multiple bacterial species were associated with the absence of toxicity, but microbes associated with toxicity were unidentifiable according to the linear discriminant analysis effect size. The nonoxidative branch of the pentose phosphate pathway was upregulated in patients with reported toxicities, indicating that metabolites from these bacterial taxa may serve as biomarkers for side effects after CD19 CAR-T-cell therapy (112). In another recent study by Hu et al., researchers investigated second-generation BCMA CAR-T-cell toxicity in patients with relapsed/refractory MM, NHL, or ALL (113). Microbiota changes were longitudinally monitored throughout CAR-T-cell therapy. Stool samples were collected prior to CAR-T-cell infusion, during CAR-T-cell infusion but prior to the development of CRS, during active CRS, and up to fourteen days after CAR-T-cell infusion. Severe CRS was associated with changes in the abundance of several gut microbiota species. As indicated by the Shannon index, there was a significantly decreased abundance of *Bifidobacteria*. Alpha diversity was observed after CAR-T-cell infusion. Furthermore, an increase in the abundance of the *Actinomyces* and *Enterococcus* genera was also suggested to be associated with an increased incidence of CRS. Overall, antibiotic exposure and subsequent alteration of the gut microbiota are associated with increased toxicity, including CRS and ICANS, and with worsened CAR-T-cell responses.

## Potential strategies for modifying the gut microbiota to enhance the therapeutic efficacy of CAR-T cells

**Figure 2:**
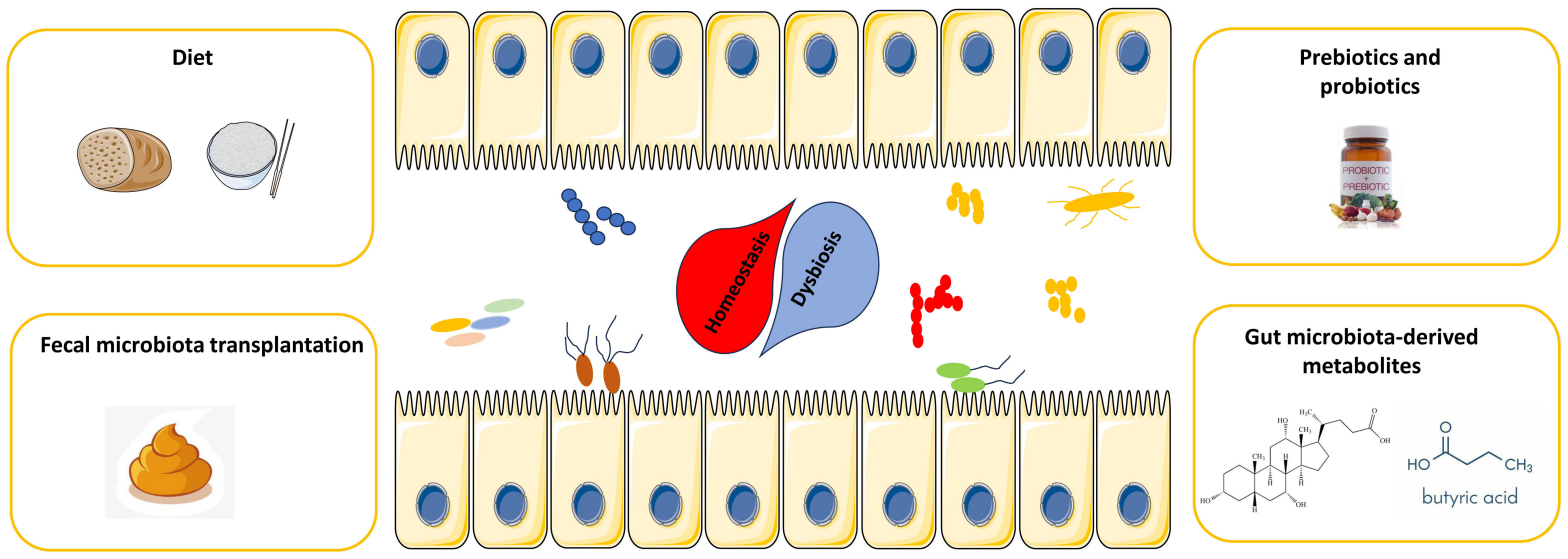
Potential strategies for harnessing the gut microbiota to potentiate CAR-T-cell efficacy while attenuating toxicity.

As mentioned above, the gut microbiota has been demonstrated to be an extrinsic factor that can predict and prospectively dictate the efficacy and toxicity of CAR-T-cell therapy, providing a novel target for improving the efficacy of CAR-T-cell therapy. Currently, several potential strategies, including FMT, administration of defined taxa and diet, have been demonstrated to improve the treatment efficacy while decreasing the toxicity of ICIs therapy through modulation of the gut microbiota. In this section, we focused on the development and future application of these potential strategies to optimize the microbiota composition to improve the efficacy of CAR-T-cell therapy and decrease the incidence of AEs (**Figure 2**).

## FMT

FMT represents the transfer of fecal microbial content from a healthy donor to the intestine of a recipient and has been widely tested in the treatment of resistant *Clostridium difficile* infection, other opportunistic infections and inflammatory bowel diseases (115, 116). Moreover, FMT has also demonstrated to improve the efficacy of a series of cancer therapies. For example, Gopalakrishnan et al. established melanoma-bearing GF mice and orally transferred the fecal microbiota of responder or nonresponder patients to anti-PD-1 therapy into these mice. Ultimately, mice treated with FMT from responder patients had significantly reduced tumor size (median tumor volume: 403.7 mm^3^ *vs*. 2301 mm^3^) and improved responses compared to those treated with FMT from nonresponder patients (109). Recently, the results of several clinical trials incorporating FMT to improve cancer therapy have been published. In a clinical trial led by Dr. Davar (NCT03341143), investigators assessed whether resistance to anti-PD-1 therapy can be overcome by changing the gut microbiota in patients with advanced melanoma (117). The patients received FMT from seven donors (including four with complete response (CR) and three with partial response (PR)) in combination with pembrolizumab treatment. The objective response rate (ORR) was 20% (3 PRs out of 15 patients), whereas the percentage of patients with durable SDs lasting >12 months was 20% (3 out of 15 patients). Another phase I clinical trial (NCT03353402) showed similar results, and the ORR in patients receiving FMT and PD-1 inhibitors was 30% (3 out of 10, including two PRs and one CR) (118). In addition to overcoming resistance to ICIs in patients with refractory cancer, FMT also showed promising efficacy in enhancing the antitumor efficacy of ICIs in first-line treatment settings in a recent study of previously untreated patients with advanced melanoma. (NCT03772988). The ORR was 65% (13 of 20, including 4 (20%) CRs), and only five patients (25%) experienced grade 3 irAEs from FMT combined with the PD-1 inhibitor. Responders experienced an enrichment of immunogenic bacteria and a loss of deleterious bacteria following FMT (119). As mentioned above, significant correlation of gut microbiome with treatment outcome and CRS grade of CAR-T therapy has been observed. Thus, it is reasonable that FMT can also help to rescued the diversity and function of gut microbiota in cancer patients. Although additional evidence is needed, combination with FMT provides a promising way to enhance the efficacy and safety of immunotherapy and may also constitute a potential strategy for enhancing the antitumor efficacy of CAR-T-cell therapy in the near future.

## Diet, prebiotics and probiotics

In addition to antibiotics and FMT, which can directly modulate the gut microbiota, diet is a critical means of regulating microbial composition and function. In recent decades, several different dietary strategies, including long-term caloric restriction, intermittent fasting, short-term starvation, high-fiber diets, ketogenic diets and fermented food, have been widely studied in the context of cancer and have been demonstrated to improve the antitumor efficacy of cancer therapy (120). Despite several limitations, dietary intervention represents a tractable strategy for modulating the function of the gut microbiota (121–125). For example, a high-fiber diet has been demonstrated to be associated with a better response and survival rate when treated with ICIs in preclinical models and in clinical cohorts and was considered to be correlated with enhanced T-cell activation and monocytic reprogramming within the TME (126). In addition, caloric restriction can promote memory T cell accumulation in the bone marrow and enhance T cell immunity to bacterial infection and tumors in T cell adoptive transfer models (127). The ketogenic diet has represented another popular diet in recent years. Ketone body 3-hydroxybutyrate can induce T cell immunity, thereby enhancing the antitumor effect of anti-PD-1 therapy in *in vivo* mice model (128). In addition to diet modulation, several naturally occurring soluble fibers, such as inulin and pectin, can be found in many vegetables and fruits. These fibers are called prebiotics and cannot be digested by gastrointestinal enzymes but can be fermented by bacteria (129, 130). Recently, prebiotics have been shown to affect the functional status of the gut microbiota in preclinical models. For example, oral inulin-gel treatments increase the abundance of short-chain fatty acid (SCFA)-producing microorganisms and amplify the antitumor efficacy of anti-PD-1 therapy (131). Pectin feeding can also enhance the ability of mice transplanted with the fecal microbiota of a patient with cancer to respond to anti-PD-1 therapy by expanding the fiber-fermenting and SCFA-producing microbiota (132). All these data suggested that traditional diet modulation or dietary components, such as prebiotics, may represent a promising way to improve immunotherapy responsiveness by targeting the microbiota.

The concept of probiotics, which refers to the specific transplantation of single microbial species and/or designer microbial consortia to enhance the response to ICIs and other forms of cancer treatment, has also emerged as an important research field (133). In 1993, a multicenter, randomized controlled study of 223 patients with uterine cervix carcinoma treated with a combination of heat-killed *Lactobacillus casei* strains (LC9018) and radiation therapy revealed that tumor regression was enhanced through the induction of an immune response against cancer cells (134). Since then, the potential applications of combination probiotics/cancer treatment have sparked interest in future studies. For example, in a small, open-label trial, 58% of patients with metastatic renal cell carcinoma (RCC) treated with CBM588 (a formulation that includes a strain of *Clostridium butyricum*) in combination with ICIs responded to treatment, whereas 20% of patients received only ICIs (135). Additionally, PFS was significantly prolonged in CBM588-treated patients to 12.7 months compared with 2.5 months in patients receiving ICB alone, thus highlighting that the addition of bifidogenic bacterial products can improve the clinical outcome of patients with RCC. Despite several limitations, there are tremendous opportunities to develop informed, ‘next-generation’ probiotics, as recent studies suggest that specific microorganisms in the gut may enhance antitumor immune responses in part through the induction of highly therapeutic TLSs in the TME, which have been favorably associated with patients’ response to ICIs across cancer types (136, 137). Together, these lines of investigation open up new possibilities for transplanting and targeting specific therapeutic microorganisms and can also be applied to enhance the antitumor efficacy of CAR-T cells in the future.

## Targeting gut microbiota-derived metabolites

Gut microbiota-derived metabolites represent a variety of small molecules produced or transformed by intestinal microorganisms that not only exert direct effects in the intestine but also modulate the function of cells in remote organs. Unlike gut bacteria, which are predominantly located in the luminal compartment of the intestine, gut microbiota-derived small molecules can easily cross the epithelial layer and diffuse through the entire circulation (138). It is estimated that 5 and 10% of all plasma metabolites are derived from the gut microbiota; however, these products generated by the gut microbiota were considered merely dead-end byproducts of their metabolic pathways for a long time (139, 140). In the past decade, gut microbiota-derived metabolites have received increasing attention in cancer research (141–143). For example, short-chain fatty acids (SCFAs), which are synthesized by the bacterial fermentation of dietary fiber, are the most abundant class of microbial metabolites and are composed of carboxylic acids with aliphatic tails of 1-5 carbons (144). Bacterial SCFAs, such as acetate (C2), propionate (C3), butyrate (C4) and valerate (C5), play key roles in regulating anticancer immunity and cancer immune surveillance (145). Butyrate reportedly enhances the therapeutic efficacy of anti-PD-1 agents by increasing CD4+ and CD8+ T-cell infiltration in the TME in tumor-bearing mice humanized with intestinal microbes from colorectal cancer (CRC) patients (132). Moreover, replenishing butyrate prior to anti-PD-1 treatment was sufficient to improve the therapeutic efficacy in nonresponders. Similarly, He and colleagues indicated that the SCFA butyrate could directly potentiate the antitumor CD8+ T-cell response via ID2-dependent IL-12 signaling, suggesting the potential beneficial role of butyrate supplementation in anticancer immunity therapy (146). In a study directed by Luu et al, microbiota-modulated SCFA, may also enhance the antitumor action of cytotoxic lymphocytes and CAR-T cells through metabolic and epigenetic remodeling of CAR-T cells. Mechanically, they found that *in vitro* treatment of CTLs and CAR-T cells with pentanoate and butyrate increases the function of mTOR as a central cellular metabolic sensor, and inhibits class I histone deacetylase activity, which increased expression of effector molecules such as CD25, IFNγ, and TNFα in syngeneic murine melanoma and pancreatic cancer models (147, 148). In addition to SCFA, Other microbial-derived metabolites, signaling through aryl hydrocarbon receptors (AhR), may also play a role in contributing to CD8+ T cell exhaustion by upregulating inhibitory receptors and downregulating cytokine production, thereby altering the ability of T cells to kill tumor cells. Supplementation or inhibition of such microbially secreted bioactive metabolites may potentially be used to reinvigorate the immune response (149, 150). Taken together, gut microbiota-derived metabolites represent another promising target to enhance the efficacy and safety of CAR-T-cell therapy in the future.

## Current challenges and future perspectives

In the past decade, immunotherapy has made significant progress and has gradually become the most important treatment for tumors. CAR-T-cell therapy represents the most promising treatment option for cancer, and a series of CAR-T-cell products have been approved for the treatment of R/R B-cell leukemia or lymphoma. Although promising in terms of efficacy, several limitations still exist, including poor efficacy in solid tumors and toxicity, such as CRS and ICAN. In contrast to the stability of the human genome, the modifiable nature of the gut microbiota renders it a promising opportunity for CAR-T-cell therapy. Recently, the role of the gut microbiota in cancer therapy has been established, and growing evidence has suggested that targeting the gut microbiota is a promising method for enhancing the antitumor efficacy of CAR-T-cell therapy. As mentioned above, FMT, microbiota-derived metabolites, diet, prebiotics and probiotics have been explored in several studies and are regarded as effective strategies for enhancing the antitumor efficacy of CAR-T cells through modifying the composition and function of the gut microbiota.

Although it is believed that modulating the gut microflora will likely be a promising method for managing CAR-T-cell therapy, several potential challenges should be mentioned. First, most of the evidence supporting the relationship between the microbiota and immunotherapy has been obtained from mouse models. As the gut microbiota in mice is not identical to that in humans and the innate and adaptive immune systems of mice are also different from those of humans, a deeper comprehension of the underlying molecular mechanisms and additional humanized animal models or clinical trials are needed to further explore the underlying correlation. Second, the exact mechanisms underlying the correlation between the gut microbiota and cancer genesis/therapy have not been fully characterized, and evidence of causation between them is still lacking. However, further *in vitro* and *in vivo* studies are needed to determine the causal mechanisms involved. In the context of CAR-T-cell therapy, transferring defined microbiota constituents from responders and nonresponders into GF cancer mice would enable us to elucidate the causal contribution of the gut microbiota and its bioactive metabolites. Interindividual microbiota variability represents another formidable challenge in identifying the impacts of CAR-T-cell therapy and AEs on the gut microbiota and their bioactive metabolites. Indeed, the microbiota populations of CAR-T-cell-treated patients reported by Smith et al. and Hu et al. are quite different (27, 113). In different trials, varying taxa have been associated with impaired immunotherapy responsiveness, indicating that defining a generalizable CAR-T-cell therapy optimizing the microbial signature will be difficult. Thus, multicentric clinical trials with high-quality training and validation sets for identifying CAR-T-cell therapy-related gut microbiota are urgently needed. Moreover, artificial intelligence technologies can be used in this process. Fecal microbiota transfer into CAR-T-cell-resistant individuals may optimize responsiveness and even lead to the conversion of nonresponders to responders. However, as a living body, such treatment can pose risks of introducing potentially harmful bacteria into patients who are already severely immunocompromised, and it is difficult to guarantee its safety. Thus, identification and isolation of defined microbiota that mediate such favorable effects may offer a safer, more reproducible, and universal treatment option. Another challenge is the complex physiological conditions (such as gastric acid and diverse enzymes) that might digest or deactivate microbial agents before they reach the action site. Thus, the appropriate delivery route and dose should also be optimized.

## Conclusions

CAR-T-cell therapy has revolutionized the history of cancer treatment. However, several disadvantages, such as low efficacy and high toxicity, limit the widespread application of CAR-T cells in solid tumors. Recently, an increasing number of studies have highlighted the key role of the gut microbiota in affecting the efficacy of cancer therapies and their side effects. Similarly, overwhelming preclinical and clinical evidence also supports a critical role of the gut microbiota in the response to and toxicity of CAR-T-cell therapy, which indicates that modulating the gut microbiota is a promising therapeutic strategy for enhancing the antitumor efficacy and attenuating the toxicity of CAR-T-cell therapy. Currently, research on microbial therapy for cancer is still in its infancy, and further mechanistic dissection via cellular and animal studies as well as validation with larger longitudinal clinical cohorts are needed. Despite these challenges, targeting the gut microbiota remains a promising strategy for improving CAR-T-cell therapy in the future.

## Author contributions

P-FZ: Conceptualization, Funding acquisition, Investigation, Methodology, Writing – original draft, Writing – review & editing. DX: Conceptualization, Investigation, Methodology, Writing – original draft.
